# Application of a modified lung ultrasound score in assessing the severity of neonatal pneumonia: a multicenter prospective study

**DOI:** 10.3389/fmed.2025.1711107

**Published:** 2025-12-19

**Authors:** Jiang Cheng, Hui Ge, Yunlu Huang

**Affiliations:** 1 Department of Ultrasound, The Third People’s Hospital of Bengbu, Bengbu, China; 2 Affiliated Suzhou Hospital of Nanjing University Medical School, Suzhou, China

**Keywords:** neonatal pneumonia, lung ultrasound scoring, diagnostic value, risk factors, prognosis evaluation

## Abstract

**Objective:**

To evaluate a modified twelve-zone lung ultrasound (LUS) scoring system for diagnosing neonatal pneumonia and assessing disease severity.

**Methods:**

In this multicenter prospective study (January 2023–02tober 2024), 160 neonates with pneumonia underwent both LUS and chest X-ray. Diagnostic performance (AUC, sensitivity, specificity) was compared, and correlations between LUS indicators (B-lines, consolidation area, pleural line abnormalities) and clinical outcomes (respiratory distress scores, PaO_2_/FiO_2_ ratio) were analyzed using multivariate regression.

**Results:**

LUS demonstrated superior diagnostic accuracy to X-ray (AUC: 0.93 vs. 0.83; sensitivity 94.0 vs. 78.5%, specificity 88.5 vs. 78.5%, both *P* < 0.05). B-line reduction correlated with improved respiratory distress scores (*r* = 0.856, *P* < 0.001), and consolidation area reduction correlated with PaO_2_/FiO_2_ ratio improvement (*r* = 0.801, *P* < 0.001). Multivariate analysis identified B-line count (OR = 1.154), consolidation area (OR = 2.962), pleural line abnormalities (OR = 6.408), and CRP (OR = 1.102) as independent severity predictors (*P* < 0.05).

**Conclusions:**

The modified twelve-zone LUS system accurately diagnoses neonatal pneumonia, quantifies severity, and dynamically monitors treatment efficacy, offering a radiation-free alternative to X-ray for clinical practice.

## 1 Introduction

Neonatal pneumonia is one of the most common and severe respiratory infections during the neonatal period, marked by high morbidity and mortality rates (1, 2). Owing to the immaturity of their immune systems, neonates have weak resistance to pathogens, leading to rapid progression of infections. However, the clinical manifestations are often atypical—such as respiratory distress and feeding difficulties—and lack specificity (3, 4). Early and accurate diagnosis is crucial for timely intervention, reducing mortality, and improving prognosis, yet it remains a significant challenge.

Traditional diagnostic methods for neonatal pneumonia mainly rely on chest X-rays, but these have several limitations. First, X-ray examinations involve ionizing radiation, which may potentially affect the growth and development of neonates (5, 6). Second, X-rays have low sensitivity and specificity for detecting early and subtle lesions, leading to missed or incorrect diagnoses (7–9). Additionally, performing X-rays at the bedside in real time is challenging, making it difficult to meet the diagnostic and treatment needs of critically ill neonates. Therefore, there is an urgent need for a safe, convenient, and accurate diagnostic method.

Lung ultrasound scoring (LUS), as a radiation-free, repeatable, and bedside-operable imaging method, has gained widespread attention in pediatrics in recent years, especially in neonatal intensive care units (10, 11). The bilateral twelve-zone lung ultrasound scoring system has shown promising results in evaluating neonatal pneumonia, effectively reflecting the extent and severity of pulmonary lesions (11, 12). However, existing methods still have shortcomings in the uniformity of scoring standards, operational simplicity, and objectivity of results, which limit their clinical application. This study aims to improve the bilateral twelve-zone lung ultrasound scoring method and explore its value in assessing the severity of neonatal pneumonia. By optimizing the scoring system, we hope to enhance the accuracy and repeatability of ultrasound evaluations, providing reliable diagnostic evidence for clinical practice and aiding in the early identification and precise treatment of neonatal pneumonia.

## 2 Materials and methods

### 2.1 Study design

This study adhered to the STARD guidelines for diagnostic accuracy studies. This multicenter, prospective study aimed to evaluate the value of a modified twelve-zone lung ultrasound scoring system in assessing the severity of neonatal pneumonia. Conducted from January 2023 to October 2024, the research took place in the neonatal departments of Bengbu Third People’s Hospital and Suzhou Hospital affiliated with Nanjing University Medical School. The study adhered to the principles of the Declaration of Helsinki and received approval from the hospital ethics committee [Ethics Approval No. (2023) s2]. Informed consent was obtained from the parents or guardians of all participating infants.

### 2.2 Study population

**Inclusion criteria:** Neonates within 28 days of birth; clinically suspected pneumonia with at least one of the following symptoms: dyspnea, fever, cough, tachypnea, or cyanosis; signed informed consent obtained from parents or guardians. In this study, the final diagnosis of neonatal pneumonia was made by the attending neonatologist according to predefined criteria combining acute respiratory symptoms and signs (tachypnea, increased work of breathing, crackles on auscultation) with compatible imaging findings on chest radiograph and/or lung ultrasound, especially the presence of focal or multifocal consolidations. Infants with clinical and imaging features typical of viral bronchiolitis (predominant wheezing, diffuse bilateral interstitial changes or hyperinflation without focal consolidation) were diagnosed as bronchiolitis and were not included in this cohort. Microbiological cultures and virological tests of respiratory samples were performed when clinically indicated but were not obtained systematically in all infants.

**Exclusion criteria:** Presence of congenital heart disease, congenital pulmonary developmental anomalies, or other severe organic diseases; prior lung surgery or invasive procedures; participation in other clinical trials that could affect the outcomes of this study; refusal by parents or guardians to participate. Before enrollment, all infants with suspected pneumonia underwent transthoracic echocardiography performed by a pediatric cardiologist to systematically exclude structural congenital heart disease, including atrial or ventricular septal defects and patent ductus arteriosus with hemodynamically significant left-to-right shunts that could cause interstitial pulmonary congestion.

A total of 160 neonates meeting the criteria were enrolled, comprising 88 males (55.0%) and 72 females (45.0%). The average gestational age was 38.5 ± 1.2 weeks, and the mean birth weight was 3,200 ± 250 g. All infants developed respiratory symptoms within 7 days after birth.

### 2.3 Examination methods

#### 2.3.1 Ultrasound examination methods

Neonatal lung ultrasounds were performed using a Canon Aplio 300 ultrasound diagnostic system equipped with a 12 MHz linear array probe. Infants were placed in supine and lateral positions. Using the parasternal, anterior axillary, and posterior axillary lines as vertical landmarks, each hemithorax was divided into anterior (between the parasternal and anterior axillary lines), lateral (between the anterior and posterior axillary lines), and posterior regions (posterior to the posterior axillary line, extending to the paravertebral area). A horizontal line connecting both nipples further subdivided each region into upper and lower zones; thus, each lung was divided into six zones (anterior upper, anterior lower, lateral upper, lateral lower, posterior upper, and posterior lower), for a total of 12 zones bilaterally. A high-frequency probe was placed perpendicular to the ribs and parallel to the intercostal spaces, scanning from the second intercostal space downward, moving from top to bottom and from medial to lateral. Ultrasound images were recorded and stored.

Consolidation area was measured by two independent sonographers on a frozen two-dimensional image showing the largest cross-sectional section of each lesion. Maximal longitudinal and perpendicular diameters were obtained using electronic calipers, and the area was approximated as length × depth (cm^2^). Interobserver reliability for consolidation area measurements was assessed using the intraclass correlation coefficient (ICC) based on 20% of cases randomly selected for duplicate readings.

#### 2.3.2 Modified bilateral twelve-zone lung ultrasound scoring

The modified bilateral twelve-zone LUS score used in this study was developed by our research team specifically for neonatal pneumonia, based on the classical twelve-zone LUS scoring system (0–3 points per lung zone; maximum 36 points) that has been reported in previous neonatal studies. To better capture the full spectrum of lung involvement in this population, we expanded the per-zone scale to 0–7 points and explicitly integrated both aeration patterns (A- and B-lines) and the anatomical extent of consolidation, thereby increasing the dynamic range of the score and improving discrimination between intermediate severity levels and small changes during treatment. The design of this score was informed by previously validated neonatal and pediatric LUS scores proposed by Brat et al. (13), Liu et al. (14), and Corsini et al. (15), but uses a broader per-zone range and more granular weighting of consolidation extent; the 0–7 per-zone weighting (maximum 84 points) was pre-specified based on expert consensus rather than derived from this cohort, in order to avoid data-driven overfitting.

Scores overfitting.derto the classical aeration patterns used in previously validated neonatal and pediatric LUS scores (predominant A-lines to coalescent B-lines), whereas scores 4nt Bpredominant Atterns used in previously validtion extent (< 1, 1res 4nt Bpre > 3 intercostal spaces). These consolidation categories and their weights were pre-specified by expert consensus of the study neonatologists and sonographers, rather than being empirically derived from this cohort.

**Scoring criteria:**

**FIGURE 1:**
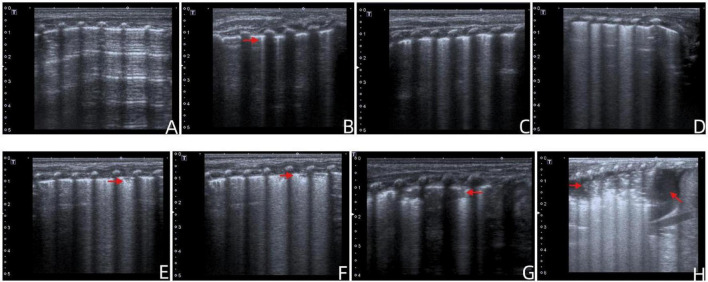
Representative lung ultrasound images for each category of the modified twelve-zone LUS score (0–7 points). **(A)** Female neonate, 13 days old. Predominantly A-lines with fewer than three isolated B-lines (score 0). **(B)** Male neonate, 10 days old. Right anterior upper lung zone with more than three non-confluent B-lines (score 1). **(C)** Male neonate, 21 days old. Left upper lung zone showing dense, partially fused B-lines (score 2). **(D)** Female neonate, 11 days old. Right lower lung zone with completely fused B-lines (score 3). **(E)** Male neonate, 19 days old. Right anterior lower lung zone with a small subpleural consolidation involving less than one intercostal space (score 4). **(F)** Female neonate, 22 days old. Left lower lung zone with consolidation involving 1–2 intercostal spaces (score 5). **(G)** Female neonate, 16 days old. Left posterior lower lung zone with consolidation involving 2–3 intercostal spaces (score 6). **(H)** Male neonate, 17 days old. Right posterior lower lung zone with consolidation involving more than three intercostal spaces accompanied by pleural effusion (score 7).

1. Predominantly A-lines with fewer than three isolated B-lines: 0 points;
2. More than three B-lines without fusion: 1 point;
3. Dense, partially fused B-lines: 2 points;
4. Completely fused B-lines: 3 points;
5. Lung consolidation involving less than one intercostal space: 4 points;
6. Lung consolidation involving 1than being empirically derived;
7. Lung consolidation involving 2than being empirically derived;
8. Lung consolidation involving more than three intercostal spaces: 7 points;

Each lung zone was scored based on the most severe finding, with scores ranging from 0 to 7 per zone, for a total possible score of 84 points. Representative ultrasound images corresponding to each scoring category (0–7 points) are shown in Figure 1.

Pleural line thickening was defined as a thickness exceeding 1 mm, measured perpendicularly from the hyperechoic pleural surface to the subpleural parenchyma using the ultrasound machine’s caliper tool. This threshold was derived from normative neonatal pleural line measurements under high-frequency probes, as reported in prior studies (16).

Irregularity: loss of smooth, hyperechoic linear appearance with focal discontinuities or undulations.

Interruption: complete break in the pleural line continuity.

Abnormalities were scored as present (1 point) or absent (0 points) per lung zone.

#### 2.3.3 Chest X-ray imaging

Chest radiographs were obtained using a United Imaging uDR370i diagnostic X-ray system set at 40 kV and 2 mAs. Infants were positioned supine for bedside imaging, with appropriate radiation shielding applied to non-exposed areas. For the purpose of this study, lung ultrasound and chest radiographs were interpreted by experienced sonographers and pediatric radiologists who were blinded to the patients who were blinded lutory data, and they were not informed of the therapeutic decisions or clinical outcomes at the time of image interpretation.

When interpreting consolidations on lung ultrasound in conjunction with chest radiographs, lesions were considered consistent with pneumonia if they were non-gravity-dependent, patchy or wedge-shaped and showed air bronchograms without obvious volume loss, whereas strictly gravity-dependent, homogeneous plate-like opacities with clear volume loss and associated displacement of fissures or mediastinal structures were interpreted as more likely to represent atelectasis and were recorded separately in the imaging report.

### 2.4 Data collection

Data collected included the following: (1) Clinical Symptoms and Signs: Fever, cough, dyspnea, groaning, nasal flaring, and intercostal retractions. (2) Laboratory Tests: Complete blood count (white blood cell count, neutrophil percentage), C-reactive protein (CRP), and blood gas analysis (PaO_2_/FiO_2_ ratio, etc.). (3) Imaging Studies: Chest X-rays were independently interpreted by two senior radiologists. In cases of disagreement, a third radiologist was consulted to reach a consensus. The ultrasound and X-ray findings were compared to analyze differences in lesion detection rate, extent, and characterization.

### 2.5 Evaluation criteria

The severity of dyspnea was assessed using the Silverman-Anderson respiratory distress scoring system, which ranges from 0 to 10 points. Higher scores indicate more severe conditions (16, 17). Based on the scoring results, patients were classified as follows: mild pneumonia (0–3 points), moderate pneumonia (4–6 points), and severe pneumonia (7–10 points). Neonatal pneumonia severity was further categorized into “severe pneumonia” (score ≥ 7 points) and “non-severe pneumonia” (score < 7 points) according to predetermined cutoff values. The modified LUS score was prospectively applied to categorize severity (mild/moderate/severe) and analyzed against clinical outcomes (e.g., length of stay, need for ventilation) using multivariate regression models.

In all participating centers, antibiotic therapy for neonatal pneumonia followed a unified institutional protocol based on contemporary national guidelines. Empirical intravenous broad-spectrum β-lactam antibiotics, with or without β-lactamase inhibitors, were initiated in all infants and adjusted according to culture results and clinical response, with a planned treatment duration of approximately 10–14 days. Respiratory support was delivered according to a common stepwise algorithm. Supplemental oxygen was provided for mild respiratory distress; high-flow nasal cannula (HFNC) was initiated for persistent tachypnea or moderate distress; continuous positive airway pressure (CPAP) or non-invasive ventilation (NIV) was used for more severe distress; and invasive mechanical ventilation (MV) was reserved for respiratory failure, recurrent apnea, or refractory hypoxemia. Ventilatory settings, including positive end-expiratory pressure, were titrated according to the same clinical targets (oxygen saturation and blood gas values) at both centers, and lung ultrasound examinations were performed under stable ventilatory conditions.

### 2.6 Statistical analysis

Statistical analyses were performed using IBM SPSS Statistics 26.0 software. Measurement data conforming to a normal distribution are expressed as mean ± standard deviation (Mean ± SD). An independent samples *t*-test was used for comparisons between groups, and a paired samples *t*-test was used for pre- and post-treatment comparisons. Count data are presented as frequencies and percentages, analyzed using the chi-square (χ^2^) test. Pearson correlation coefficients were employed for correlation analyses. Before performing correlation analyses, the distributions of continuous variables and change values (Δ) were assessed using the Shapiro variables and chspection of histograms and Qssed using the ShapirΔrespiratory distress score and Δoxygenation-related variables showed approximately normal distributions and linear relationships, Pearson correlation coefficients were used.

Variables with *P* < 0.05 in univariate analyses were included in a multivariate logistic regression model, utilizing a stepwise regression method to identify independent factors influencing the severity of neonatal pneumonia. For multivariable logistic regression models, multicollinearity among covariates was assessed using variance inflation factors (VIF), and variables with VIF < 2 were considered acceptable. Receiver operating characteristic (ROC) curves were plotted to calculate the area under the curve (AUC), comparing the diagnostic efficacy of the modified ultrasound scoring method and chest X-rays. The DeLong test was used to compare differences in AUC between groups. Using the comprehensive clinical diagnosis as the gold standard, the sensitivity, specificity, positive predictive value (PPV), and negative predictive value (NPV) of the two examination methods were calculated. In addition, receiver operating characteristic (ROC) curves of the baseline LUS score were constructed to evaluate its ability to predict the need for mechanical ventilation and prolonged hospitalization (defined as a length of stay ≥ 14 days). The optimal cut-off value was determined by maximizing the Youden index (sensitivity + specificity − 1), and the sensitivity, specificity, PPV, and NPV for this threshold were calculated. Sensitivity and specificity were compared using the McNemar test, while PPV and NPV were compared using the *Z*-test for two independent sample proportions. Time-to-event outcomes (duration until mechanical ventilation and length of hospital stay) were analyzed using multivariate Cox proportional hazards models, with baseline LUS score as a continuous covariate. There were no missing data for the main baseline variables, LUS scores, or outcomes; therefore, all analyses were performed on complete cases without imputation.

## 3 Results

### 3.1 Baseline characteristics of patients

A total of 160 neonates with pneumonia were enrolled in this study, comprising 88 males (55.0%) and 72 females (45.0%). The average gestational age was 38.5 ± 1.2 weeks, and the mean birth weight was 3,200 ± 250 g. All patients developed respiratory symptoms within 7 days after birth. The severity assessment of neonatal pneumonia showed 50 cases (31.3%) of mild pneumonia, 58 cases (36.3%) of moderate pneumonia, and 52 cases (32.5%) of severe pneumonia. Interobserver reliability for consolidation area measurements was excellent (ICC = 0.921, 95% CI: 0.850ver reliability for % of cases randomly selected for duplicate readings.

### 3.2 Comparison with chest X-rays

The concordance between ultrasound and chest X-rays in diagnosing neonatal pneumonia was 86.7%, with a Kappa value of 0.73 (*P* < 0.001), indicating good agreement between the two methods.

### 3.3 Diagnostic performance metrics

The sensitivity, specificity, positive predictive value (PPV), and negative predictive value (NPV) of ultrasound and chest X-ray in diagnosing neonatal pneumonia are presented in Table 1. Statistical analysis showed that the sensitivity, specificity, PPV, and NPV of ultrasound examination were significantly higher than those of chest X-ray examination (*P* < 0.05).

**TABLE 1:** Diagnostic performance of ultrasound and chest X-ray in neonatal pneumonia.

Examination methods	Sensitivity (%)	Specificity (%)	PPV (%)	NPV (%)
Lung ultrasonography	94.0	88.5	88.5	91.0
Chest X-ray	78.5	78.5	84.0	76.5
χ^2^/*Z*-value	10.523	8.456	2.347	2.104
*P*-value	0.001	0.004	0.019	0.035

Sensitivity and specificity were compared using the McNemar test. PPV and NPV were compared using the *Z*-test for two independent sample proportions.

### 3.4 ROC analysis

**FIGURE 2:**
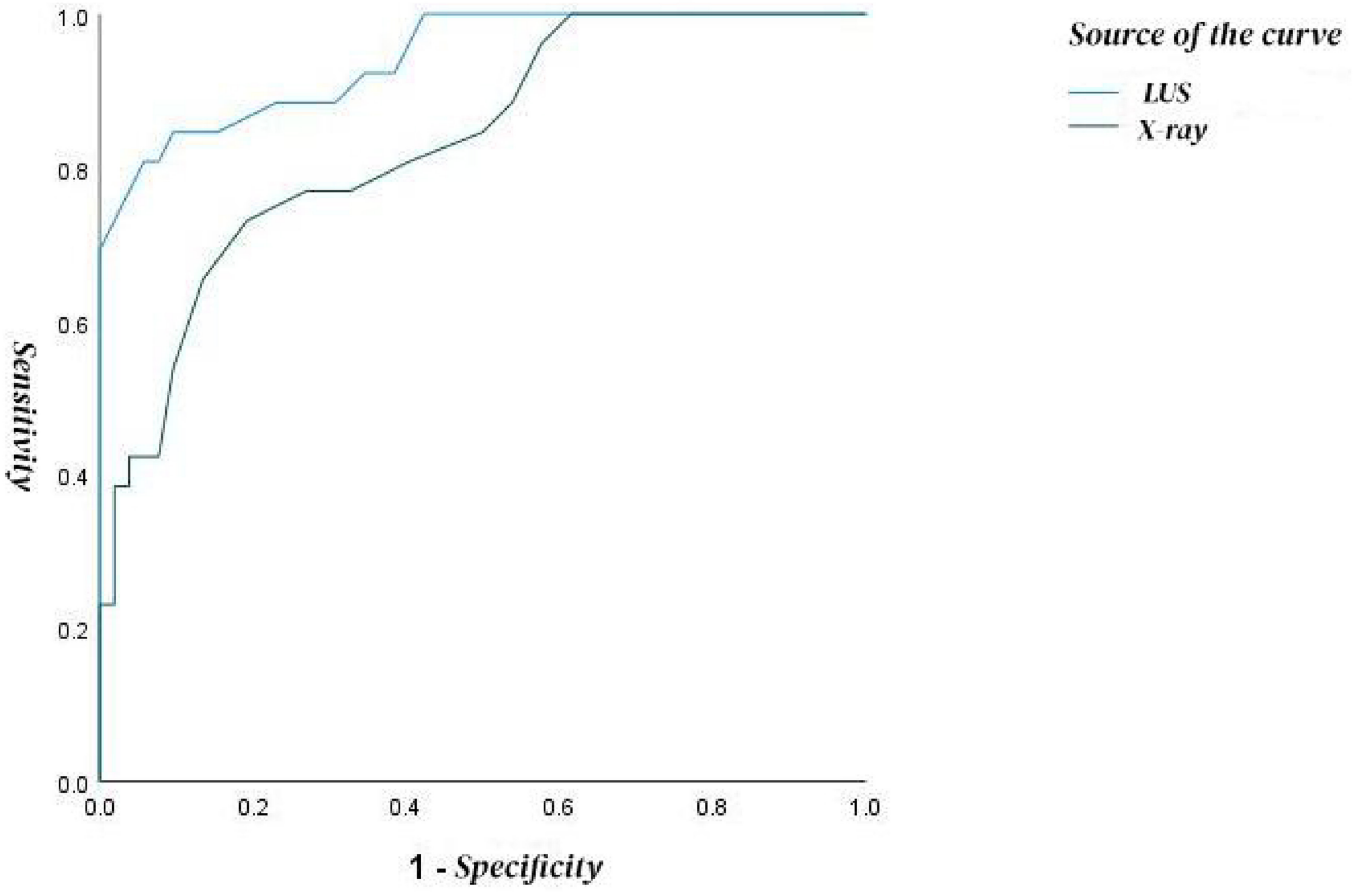
Diagnostic performance of lung ultrasound and chest X-ray for neonatal pneumonia.

The results showed that the area under the curve (AUC) for lung ultrasound in diagnosing neonatal pneumonia was 0.93 (95% CI: 0.89–0.98), while the AUC for chest X-ray was 0.84 (95% CI: 0.76–0.90), as shown in Figure 2. Statistical analysis indicated a significant difference between the AUC values of the two examination methods (*Z* = 3.00, *P* = 0.003), suggesting that lung ultrasound has higher diagnostic efficacy in neonatal pneumonia.

### 3.5 Ultrasound evaluation of treatment efficacy

After 14 days of treatment, ultrasound re-examinations were performed. The patients showed a significant reduction in the average number of B-lines, average consolidation area, and LUS scores compared to pre-treatment levels (*P* < 0.05), as shown in Table 2.

**TABLE 2:** Comparison of ultrasound findings before and after treatment in pediatric patients.

Parameters	Before treatment	After treatment	*t*-value	*P*-value
Mean number of B-lines (lines)	10.0 ± 4.0	3.0 ± 0.5	22.608	<0.001
Mean consolidated area (cm^2^)	1.8 ± 0.8	0.7 ± 0.2	18.228	<0.001
LUS score	20.1 ± 2.6	1.2 ± 0.3	92.707	<0.001

### 3.6 Correlation between ultrasound improvement and clinical efficacy

**FIGURE 3:**
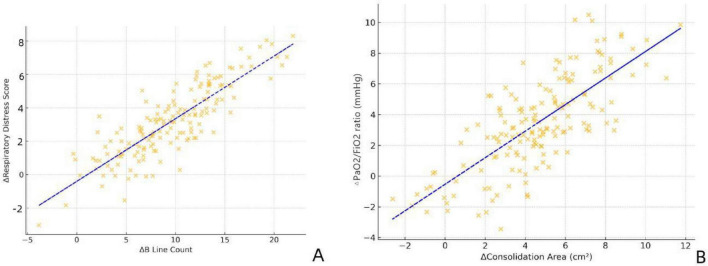
**(A)** Correlation between the change in respiratory distress score (ΔSilvermanss score (score; follow-up minus baseline, range - 10 to + 10) and the reduction in B-lines. **(B)** Correlation between the change in consolidation area and the PaO_2_/FiO_2_ ratio (P/F ratio).

Pearson correlation analysis demonstrated a close association between the degree of ultrasound improvement and clinical outcomes. There was a significant positive correlation between the reduction in B-line count and the decrease in respiratory distress scores (*r* = 0.856, *P* < 0.001). Similarly, Pearson correlation analysis demonstrated a close association between the change in consolidation area and the PaO_2_/FiO_2_ (P/F) ratio (*r* = 0.801, *P* < 0.001) (Figures 3A,B).

### 3.7 Correlation between modified LUS score and disease severity/prognosis

To validate the clinical relevance of the modified scoring system, we analyzed its correlation with predefined severity categories (mild/moderate/severe pneumonia) and prognostic indicators. Severity stratification: The median LUS score increased significantly across severity groups: Mild pneumonia: (8.2 ± 2.1); Moderate pneumonia: (15.6 ± 3.4); Severe pneumonia: (28.5 ± 4.8) (*P* < 0.001).

Prognostic relevance: Higher baseline LUS scores predicted longer hospitalization (*r* = 0.856, *P* < 0.001). On ROC analysis, a baseline LUS score of > 20 points was identified as the optimal cut-off for predicting the need for mechanical ventilation. At this threshold, the sensitivity was 80.0% and the specificity was 73.8%, with a positive predictive value of 84.6% and a negative predictive value of 88.9% for mechanical ventilation. For predicting prolonged hospitalization (length of stay ≥ 14 days), the same cut-off yielded a sensitivity of 60.0% and a specificity of 87.5%, with corresponding PPV and NPV of 80.0 and 71.4%, respectively. Scores > 20 points were associated with an increased risk of requiring mechanical ventilation (OR = 4.223, 95% CI: 2.156–8.330, *P* < 0.001). These findings confirm that the modified LUS score quantitatively reflects disease severity and predicts clinical outcomes. Multivariate Cox regression confirmed that baseline LUS scores independently predicted mechanical ventilation risk (HR = 1.326, 95% CI: 1.122–1.568, *P* < 0.001) and prolonged hospitalization (HR = 1.255, 95% CI: 1.083–1.550, *P* = 0.003).

### 3.8 Univariate analysis

Potential factors influencing the severity of neonatal pneumonia-such as demographic characteristics (gender, gestational age, birth weight), clinical indicators (CRP, white blood cell count), and ultrasound features (number of B-lines, consolidation area, pleural line abnormalities)-were compared using *t*-tests or chi-square (χ^2^) tests. A *P*-value of less than 0.05 was considered statistically significant. The results are presented in Table 3.

**TABLE 3:** Univariate analysis of factors affecting severity of neonatal pneumonia.

Variables	Severe pneumonia group (*n* = 52)	Non-severe pneumonia group (*n* = 108)	χ^2^/*t*-value	*P*-value
Sex (male/female)	30/22	58/50	0.226	0.635
Gestational age (weeks)	38.4 ± 1.1	38.6 ± 1.2	1.014	0.312
Birth weight (g)	3,100 ± 200	3,250 ± 250	3.781	<0.001
WBC (× 10^9^/L)	12.0 ± 3.0	11.5 ± 2.5	2.218	0.028
C-reactive protein (mg/L)	18.0 ± 4.0	9.0 ± 2.5	17.390	<0.001
Mean number of B-lines (lines)	15.5 ± 3.0	8.5 ± 2.6	15.160	<0.001
Mean consolidated area (cm^2^)	3.0 ± 0.7	1.0 ± 0.4	22.951	<0.001
Pleural line abnormality *n* (%)	46 (88.5%)	27 (25.0%)	56.981	<0.001
PaO_2_/FiO_2_ ratio (mmHg)	185.5 ± 25.5	275.6 ± 20.8	23.802	<0.001

### 3.9 Multivariate analysis

Variables that were statistically significant in the univariate analysis were included in a multivariate logistic regression model using the Enter method. Odds ratios (OR) and their 95% confidence intervals (CI) were calculated to identify independent risk factors influencing the severity of neonatal pneumonia. The results indicated that the number of B-lines, consolidation area, pleural line abnormalities, and CRP levels were independent risk factors affecting the severity of neonatal pneumonia (all *P* < 0.05). The PaO_2_/FiO_2_ ratio was found to be a protective factor against severe pneumonia (*P* < 0.001) (Table 4).

**TABLE 4:** Multivariate analysis of factors affecting the severity of neonatal pneumonia.

Variable	β	SE	Wald χ^2^	*P*-value	OR-value	95% CI
Number of B-lines (lines)	0.143	0.042	11.59	0.001	1.154	1.062 1.254
Consolidated area (cm^2^)	1.086	0.275	15.60	<0.001	2.962	1.722 5.096
Pleural line abnormality *n* (%)	1.857	0.493	14.18	<0.001	6.408	2.433 16.872
PaO_2_/FiO_2_ ratio (mmHg)	−0.036	0.008	20.25	<0.001	0.965	0.950 0.980
C-reactive protein (mg/L)	0.097	0.028	12.04	0.001	1.102	1.043 1.164
Constant	−6.123	1.254	23.84	<0.001	–	–

The model’s pseudo *R*^2^ value, Nagelkerke *R*^2^ = 0.86, indicates that it explains 86% of the variance in the dependent variable. The Hosmer-Lemeshow goodness-of-fit test showed χ^2^ = 7.432 with *P* = 0.384 (*P* > 0.05), suggesting a good model fit. The model’s predictive accuracy was 90.0% overall. Multicollinearity diagnostics revealed that all variance inflation factors (VIF) were less than 2, indicating no issues with multicollinearity.

### 3.10 Typical case presentation

**FIGURE 4:**
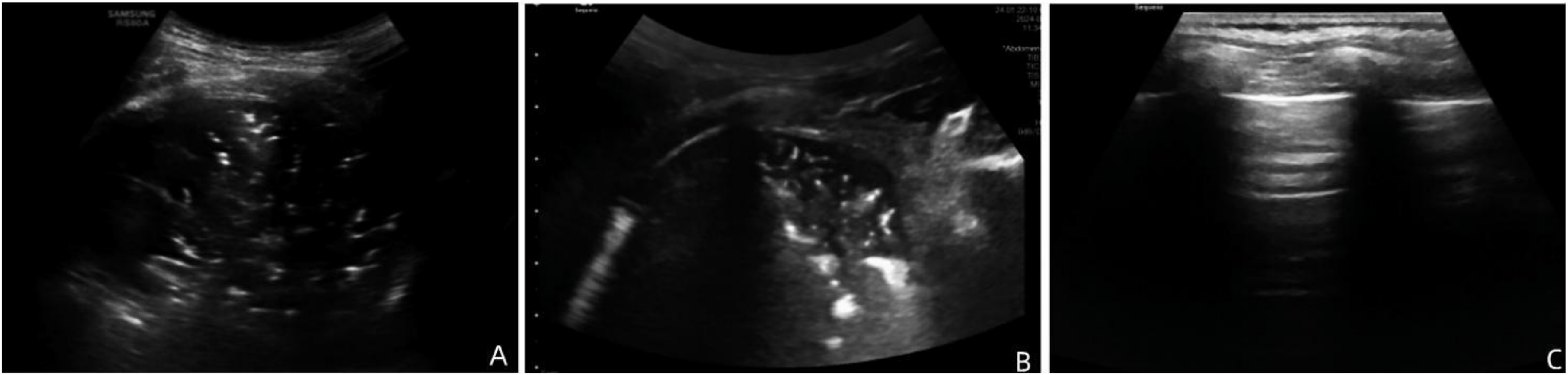
Lung ultrasound findings during the course of the disease in the pediatric patient. **(A)** LUS score of 25 before treatment; **(B)** LUS score of 13 on day 5 of treatment; **(C)** LUS score of 1 on day 14 of treatment.

**Case 1:** A full-term female infant with a birth weight of 3,100 g developed tachypnea and groaning on the 2nd day after birth, requiring oxygen support. Ultrasound examination showed a large consolidation area in the left lower lobe (approximately 3.0 cm^2^), accompanied by multiple B-lines (15 lines) and pleural line abnormalities. The LUS score was 25 points. Clinical evaluation indicated severe pneumonia with a respiratory distress score of 8. After antibiotic treatment and respiratory support, repeat LUS scores on the 3rd and 14th days were 13 and 1, respectively (Figures 4A–C).

## 4 Discussion

This study obtained important clinical findings and insights by applying a modified twelve-zone bilateral lung ultrasound scoring system to assess the severity of neonatal pneumonia. These findings suggest that the modified LUS score is closely associated with disease severity and may help identify infants at higher risk of adverse outcomes in this cohort, although its prognostic performance requires confirmation in prospective, independent studies. Multivariate logistic regression analysis demonstrated that the number of B-lines, consolidation area, pleural line abnormalities, PaO_2_/FiO_2_ ratio, CRP levels, and birth weight are independent factors influencing the severity of neonatal pneumonia. These indicators showed significant differences between the severe pneumonia group and the non-severe pneumonia group, suggesting that ultrasound findings are closely related to the clinical condition.

An increase in the number of B-lines reflects the degree of pulmonary interstitial edema or congestion, caused by fluid retention in the alveolar septa and peribronchiolar tissues (18, 19). In neonatal pneumonia, infection leads to increased pulmonary vascular permeability, resulting in fluid exudation into the interstitial space and the appearance of B-line ultrasound signs (20, 21). Our study found a strong correlation between the number of B-lines and the respiratory distress score (*r* = 0.856, *P* < 0.001), consistent with the impact of interstitial edema on lung compliance and gas exchange. Therefore, the number of B-lines can serve as a sensitive indicator for assessing the severity of dyspnea, providing clinicians with an objective evaluation tool (22).

Our modified twelve-zone LUS score demonstrated robust correlations with both severity stratification and prognosis. The progressive increase in scores across mild, moderate, and severe pneumonia categories (*P* < 0.001) validates its utility in quantifying disease burden. Furthermore, the strong association between higher baseline scores and prolonged hospitalization or need for mechanical ventilation underscores its prognostic value. These results align with prior studies (23) but extend their findings by explicitly linking the scoring system to actionable clinical endpoints, such as resource utilization and intervention thresholds.

The modified LUS score’s ability to predict outcomes (e.g., mechanical ventilation risk) highlights its potential as a dynamic tool for guiding escalation of care. For instance, a cutoff score of 20 points may serve as an early warning indicator for clinicians to initiate advanced respiratory support. This aligns with the growing emphasis on precision medicine in neonatal critical care (24), where objective imaging biomarkers complement clinical judgment.

An expansion of the consolidation area indicates an increased extent of lung consolidation, reflecting alveoli filled with exudates, inflammatory cells, and fibrin, leading to impaired gas exchange (25, 26). We also found a significant positive correlation between the reduction in consolidation area and improvement in the PaO_2_/FiO_2_ ratio (*r* = 0.801, *P* < 0.001), suggesting that changes in the consolidation area can reflect the recovery of pulmonary gas exchange function (27, 28). This finding aligns with previous studies, indicating that the degree of lung consolidation can be used to assess the severity and prognosis of pneumonia (29).

The presence of pleural line abnormalities-such as thickening, irregularity, or interruption-may be associated with alveolar structural damage, interstitial fibrosis, and pleural involvement (30, 31). These changes are more common in severe pneumonia and represent disease progression. In our study, pleural line abnormalities were confirmed as important ultrasound signs of severe pneumonia, with their incidence significantly higher in the severe pneumonia group than in the non-severe group (88.5 vs. 25.0%, *P* < 0.001). This suggests that detecting pleural line abnormalities is crucial for the early identification of critically ill infants.

Additionally, our results highlight the advantages of ultrasound in monitoring treatment efficacy. As treatment progresses, reductions in the number of B-lines and the consolidation area are closely related to the alleviation of respiratory symptoms and improvements in oxygenation function. This provides clinicians with a real-time, non-invasive monitoring tool to assess the effectiveness of therapeutic regimens and make timely adjustments.

Previous studies (32–34) have reported the value of lung ultrasound in diagnosing neonatal pneumonia, but they often used traditional scoring methods with inconsistent standards and complex procedures. In this study, we modified the bilateral twelve-zone lung ultrasound scoring system by optimizing the scoring criteria and simplifying the operational process, which enhanced the objectivity and repeatability of the scores. Our modified method demonstrated higher accuracy and practicality in assessing disease severity, further expanding the application of lung ultrasound in neonatal pneumonia. Furthermore, Zayani et al. recently compared different LUS scoring systems in infants with bronchiolitis and showed that extended scores with a broader range can improve prognostic accuracy, supporting ongoing efforts toward standardization of LUS scores across diseases (35). Our findings are also in line with the recent ESICMscoring systems in infants with bronchiolitis and shemphasizes the importance of standardized aeration scoring systems and their potential role in guiding clinical decision-making in critically ill patients (36). Moreover, a recent BMC Pediatrics study in infants with severe pneumonia showed that higher quantitative 12-zone LUS scores were associated with worse oxygenation and high-flow nasal cannula failure, supporting the use of structured LUS scoring to assess disease severity and outcomes in pediatric pneumonia (37).

Lung ultrasound offers significant advantages in detecting neonatal pneumonia, foremost among them being its exceptional early detection capability. With high resolution and real-time imaging, lung ultrasound can identify subtle structural changes in the lungs during the early stages of disease—such as pulmonary interstitial edema and small consolidations—which aids in the prompt diagnosis of neonatal pneumonia and timely initiation of treatment measures. Secondly, lung ultrasound is valuable for monitoring treatment response. Since ultrasound examinations can be repeatedly performed at the bedside, physicians can dynamically track changes in the lung lesions of infants. We found that improvements in ultrasound indicators are closely related to clinical efficacy, providing an objective basis for evaluating treatment outcomes and adjusting therapeutic plans. Finally, lung ultrasound eliminates radiation exposure associated with traditional chest X-rays or CT scans. Unlike chest X-ray examinations, lung ultrasound involves no ionizing radiation, making it safer for neonates—especially those requiring frequent imaging evaluations. This aligns with the pediatric “As Low As Reasonably Achievable” (ALARA) principle of minimizing radiation exposure (38–40). In addition, our results are consistent with the recent international consensus guidelines on the use of lung ultrasound for neonatal respiratory distress, which provide evidence-based recommendations for LUS applications in diagnosis, severity assessment, and ventilatory management, and explicitly endorse standardized aeration scoring approaches in neonates (41). However, lung ultrasound cannot reliably differentiate bacterial pneumonia from viral lower respiratory tract infections or atelectasis, because these entities may present with overlapping patterns of subpleural consolidations and B-lines. Therefore, LUS findings should be interpreted in conjunction with clinical, laboratory, and, when available, microbiological data, rather than used as a standalone etiologic diagnostic tool.

This study has certain limitations. Firstly, the sample size is relatively small, which may introduce selection bias. Although the results are statistically significant, their generalizability needs to be confirmed through larger, multicenter studies. Secondly, lung ultrasound examination is highly operator-dependent with a steep learning curve. The results can be influenced by the examiner’s experience and technical proficiency. While we provided standardized training to the ultrasound physicians involved, emphasis on operator training and credentialing remains essential in clinical practice to ensure the accuracy and reproducibility of examination results. While our study established correlations between the modified LUS score and prognosis, future studies should validate predefined score thresholds (e.g., > 20 points) as decision-making benchmarks in larger cohorts. Thirdly, only late preterm and term infants were included (mean gestational age 38.5 ± 1.2 weeks), so our findings may not be generalizable to younger or more critically ill preterm populations, in whom LUS scoring is often considered particularly relevant. In addition, we did not prospectively calculate the classical 0S scoring is often considered particularly reltherefore could not provide direct head-to-head comparative data between the modified and classical scoring systems; future multicenter studies should address this important question. Additionally, microbiological and virological investigations were not systematically performed in all infants, and diagnoses were based primarily on clinical and imaging criteria. Therefore, a certain degree of misclassification between pneumonia and other lower respiratory tract infections, particularly viral bronchiolitis in cases with near-normal inflammatory markers, cannot be completely excluded. Another limitation is that, although predefined imaging criteria were used to distinguish pneumonic consolidations from gravity-dependent atelectasis, the differentiation between these entities on bedside lung ultrasound and chest radiographs is not always clear-cut, and a certain degree of lesion-level misclassification cannot be completely excluded. Moreover, we did not include a control cohort of non-pneumonic neonates with other causes of respiratory distress, such as transient tachypnea of the newborn or respiratory distress syndrome, so the specificity and generalizability of the modified LUS score across different respiratory pathologies remain uncertain. Finally, detailed ventilator settings, including exact positive end-expiratory pressure levels and changes in respiratory support during follow-up, were not systematically recorded at the time of each lung ultrasound examination, so we cannot completely exclude some influence of treatment-related heterogeneity on the ultrasound findings.

However, because of the observational design and lack of external validation, our findings should be interpreted as hypothesis-generating rather than definitive evidence of prognostic accuracy.

Future prospective studies should therefore include parallel calculation of established neonatal and pediatric LUS scores together with the modified twelve-zone score, in order to perform head-to-head comparisons of discrimination, calibration, and clinical utility.

## 5 Conclusion

This study confirms the significant value of the modified bilateral twelve-zone lung ultrasound scoring system in assessing the severity of neonatal pneumonia. As a safe, convenient, and radiation-free imaging method, ultrasound can sensitively and accurately reflect pulmonary lesions and shows a strong correlation with clinical indicators, making it a recommended tool for routine diagnosis and treatment guidance. However, accurate interpretation requires consideration of neonatal anatomical and physiological characteristics and demands skilled operators to avoid misdiagnosis.

## Data Availability

The raw data supporting the conclusions of this article will be made available by the authors, without undue reservation.
